# Blocking hsa_circ_0074027 suppressed non-small cell lung cancer chemoresistance via the miR-379-5p/IGF1 axis

**DOI:** 10.1080/21655979.2021.1987053

**Published:** 2021-10-19

**Authors:** Shizhen Zheng, Chao Wang, Hao Yan, Yuejun Du

**Affiliations:** a Department of Infectious Disease, The Second People’s Hospital of Chengdu, Chengdu, Sichuan, China; b Department of Geriatrics International Medical Center, The Third People’s Hospital of Chengdu, Chengdu, Sichuan, China; c Department of Respiratory Disease, The Second People’s Hospital of Chengdu, Chengdu, Sichuan, China

**Keywords:** hsa_circ_0074027, chemoresistance, NSCLC, miR-379-5p, IGF1

## Abstract

Cancer cell chemoresistance is the primary reason behind cancer treatment failure. Previous reports suggest that circular RNA (circRNA) hsa_circ_0074027 (HC0074027) is a crucial modulator of non-small cell lung cancer (NSCLC) disease progression. Herein, we delineated the underlying mechanism of HC0074027-regulated chemoresistance in NSCLC. We employed quantitative real-time polymerase chain reaction (qRT-PCR) or Elisa in the detection of HC0074027, micoRNA-379-5p (miR-379-5p), and insuline-like growth factor I (IGF1) expressions. Cell survival was evaluated via the 3-(4,5-Dimethylthiazol-2-yl)-2,5-diphenyltetrazolium bromide (MTT). Direct associations among HC0074027, miR-379-5p, and IGF1 were examined via dual-luciferase reporter (DLR) and RNA immunoprecipitation (RIP) assays. Lastly, HC0074027 was incorporated into nude mice to examine its biological activity *in vivo*. Based on our analysis, HC0074027 levels strongly correlated with NSCLC chemoresistance to docetaxel (DTX), cisplatin (DDP), and paclitaxel (PTX). Alternately, HC0074027 silencing enhanced chemosensitivity *in vitro. In vivo*, HC0074027 downregulation suppressed tumor expansion and increased cancer cell sensitivity to chemotherapy. We also revealed that HC0074027 physically interacts with miR-379-5p to exert its biological function *in vitro*. Moreover, IGF1 is a functionally crucial target of miR-379-5p in modulating NSCLC chemoresistance *in vitro*. Finally, we demonstrated that HC0074027 can indirectly modulate IGF1 levels via sequestering miR-379-5p. We demonstrated that HC0074027 promotes NSCLC chemoresistance via sequestering miR-379-5p activity, and modulating IGF1 expression. Our work highlights the significance of HC0074027 in NSCLC chemoresistance and suggests HC0074027 to be an excellent candidate for targeted NSCLC therapy.

## Introduction

Non-small cell lung cancer (NSCLC) contributes to a large portion of cancer morbidity and mortality on the global stage [1]. Traditionally, NSCLC is treated with chemotherapeutic agents like docetaxel (DTX), cisplatin (DDP), and paclitaxel (PTX) [2–6]. However, chemoresistance to these drugs is a major reason of failure in the fight against NSCLC [7–9]. Given the prevalence and severity of NSCLC, it is both urgent and necessary to establish an underlying mechanism behind NSCLC chemoresistance to advance the development of targeted and effective drugs that can successfully combat this disease.

Circular RNAs (circRNAs) are circular noncoding RNAs [10–12] that have gained particular interest in the field of cancer research [13,14]. CircRNAs famously lack the 5ʹ cap and 3ʹ poly (A) tail, which are highly characteristic of messenger RNAs (mRNAs). In recent years, multiple studies demonstrated a clear link between circRNAs dysregulation and malignant tumor chemoresistance, including that of NSCLC [15–18]. Additionally, circRNAs are suggested to serve as crucial regulators of tumor progression, dissemination, and drug response in lung cancer, and are recommended indicators for lung cancer diagnosis, prognosis, and therapy [19,20]. Despite its close association with NSCLC [21–24], hsa_circ_0074027 (HC0074027)-regulated NSCLC chemoresistance is not widely studied.

Herein, we are aimed at exploring the biological role of HC0074027 in NSCLC. We examined the expressions and functions of HC0074027 and miR-379-5p in chemoresistant NSCLC cells. To achieve a complete understanding of the underlying mechanism behind HC0074027 activity, we assessed chemoresistance *in vitro* and tumor expansion *in vivo*. We hypothesized direct binding between HC0074027 and miR-379-5p, which ultimately affected the expression of Insuline-like growth factor I (IGF1). Our work highlights a new pathway of HC0074027-regulated NSCLC chemoresistance and opens up new avenues for targeted anti-NSCLC treatment.

## Materials and methods

### Patients and tissue samples

120 NSCLC patients, cured with DTX therapy, were selected from the second people’s Hospital of Chengdu from January 2014 to November 2019. Drug effectiveness was evaluated in these patients once in 2 weeks during DTX therapy. Moreover, they were followed up once in 3 months till disease progression or death. Recurrent NSCLC was defined (SolidTumors, RECIST 1.1) as a rise in lesion diameter sum by 5 mm or 20% versus baseline [25,26]. An overall of 120 tissue samples were collected, with 60 identified as primary NSCLC (sensitive) and another 60 as recurrent NSCLC (resistant) tissues. We also acquired informed written consent from all participants and received ethical approval from our institution prior to the commencement of the study.

### Establishing DTX-resistant NSCLC cells (DRNCs)

We acquired human NSCLC cell lines H1299 (Code. 0185) and A549 (Code. 0033) from (Duque deCaxias, Rio de Janeiro, Brazil). H1299 cells were grown in RPMI-1640 medium (GIBCO-BRL, GrandIsland, NY, USA), with 2 mM L-glutamine, whereas A549 cells were grown in DMEM medium (GIBCO-BRL), with 4 mM L-glutamine. H1299 and A549 cells were made DTX-resistant via continuous exposure to increasing DTX (Sigma-Aldrich, Milwaukee, WI, USA) levels. Finally, the H1299/DTX and A549/DTX cells were grown in culture medium with 10 µg/L of DTX to maintain DTX resistance. All cell cultures contained 10% of fetal bovine serum (GIBCOBRL) and were grown in an incubator with 5% CO_2_ and at 37°C.

### siRNA and other plasmid incorporation

Si-HC0074027 (corresponding control group si-NC), si-IGF1 (corresponding control group si-NC), as well as miR-379-5p–mimic (M) and – inhibitor (I) were acquired from RiboBio (Guangzhou, China) and incorporated into cells via Lipofectamine 3000 (Invitrogen, Carlsbad, USA) (siRNAs: 50 nM, miRNA M and I: 50 nM), following operational guidelines.

### RT-qPCR

Following RNA extraction and convertion into cDNA, cDNA assessment was done with a SYBR green kit (Takara, Dalian, China) and an RT-qPCR instrument (Bio-Rad Laboratories, Berkeley, USA). Relative gene levels were quantified via the 2^−ΔΔCt^ formula and HC0074027 and IGF1 mRNA was normalized to the expression of GAPDH transcript, and miR-379-5p to U6. The following primers were used in the RT-qPCR experiments: HC0074027: (F: 5ʹʹʹ-GCGTCCCTGTGTATGTTGGA-3ʹ, R: 5ʹ-GTCTGTCTTAAAGCGACAGCG-3ʹ); miR-379-5p: (F: 5ʹ-GCGCTGGTAGACTATGGAA-3ʹ, R: 5ʹ-GTGCAGGGTCCGAGGT-3ʹ);:IGF1: (F: 5ʹ-GCTCTTCAGTTCGTGTGTGGA-3ʹ, R: 5ʹ-CGACTGCTGGAGCCATACC-3ʹ); GAPDH: (F: 5ʹ-GTGGGCATCAATGGATTTGG-3ʹ, R: 5ʹ-ACACCATGTATTCCGGGTCAAT-3ʹ); U6: (F: 5ʹ-CGCTTCGGCAGCACATATAC-3ʹ, R: 5ʹ-TTCACGAATTTGCGTGTCAT-3ʹ).

### 3-(4,5-Dimethylthiazol-2-yl)-2,5-diphenyltetrazolium bromide (MTT) assay

Following siRNA/plasmid incorporation, 5,000 H1299/DTX and A549/DTX cells were plated per well of 96-well plates and exposed to DTX, DDP, and PTX for 48 h. Subsequently, 2 mg/mL of MTT reagent (Sigma-Aldrich) was introduced to cells and allowed to react for 4 h. Formazan was formed in living cells, which were then resolved in 100 µl of dimethylsulfoxide, and absorbance read at 570 nm, with a microplate reader. DTX, DDP, and PTX IC50s were computed on GraphPad Prism 7 (GraphPad Software, San Diego, USA).

### Dual-luciferase reporter (DLR) assay

MiR-379-5p docking sites harboring HC0074027 or IGF1 were cloned into psiCHECK2 (Promega, Fitchburg, WI) to generate the luciferase reporter plasmids WT-HC0074027 and WT-IGF1, respectively. We also generated the mutated forms of the above luciferase reporter plasmids (MUT-HC0074027 and MUT-IGF1). Next, the aforementioned vector, along with miRNA-NC or miR-379-5p-M, were co-incorporated into 293 T cells and incubated for 48 h. Finally, luciferase activity was measured with the DLR Assay Kit (Promega).

### RNA pull-down assay

Biotinylated miR-379-5p (bio-miR-379-5p) or bio-NC was incorporated into H1299/DTX and A549/DTX cells. After 48 h, cellular lysis was performed and streptavidin-coupled magnetic beads (Invitrogen) was introduced, before incubation for 2 h. Next, the biotin-coupled RNA complex was precipitated. Lastly, HC0074027 enrichment was assessed via RT-qPCR, after isolation of bound RNAs.

### RNA immunoprecipitation (RIP) assay

The EZ-Magna RIP ™ RNA-Binding Protein Immunoprecipitation Kit (Millipore, Billerica, MA, USA) was employed for the RIP evaluation. In short, after H1299/DTX and A549/DTX cellular lysis with RIP lysis buffer and RNase I (Millipore), 100 µL cellular lysate was exposed to RIP buffer carrying antibody-precoated magnetic beads. HC0074027 and miR-379-5p were then precipitated as described in the kit directions and their levels were assessed via RT-qPCR.

### Elisa assay

An ELISA kit (R&D Systems) and its operational guidelines were employed for the quantification of IGF1 in different culture media. We performed 3 replicates of each experiment and the average values are presented in this manuscript.

### Animal studies

Our animal protocols abided by the animal ethical review board of our hospital. 20 female BALB/c nude mice (ALF Biotechnology, Nanjing, China), aged 7-week-old, were recruited for this study and arbitrarily separated into 4 populations (n = 5/group). To achieve a subcutaneous xenograft, sh-NC or sh-circ-incorporated A549/DTX cells were administered to the right flanks of nude mice. The xenograft tumors were allowed to grow and once it reached 100 mm^3^ in size, DTX (5 mg/kg) or PBS (corresponding control group) was provided via tail vein once in every 3 days. Tumor volume (mm^3^) was monitored and recorded once a week, computed as follows: length × width^2^ × 0.5. After 28 days, the tumors were excised, weighed, and used for subsequent analysis.

### Statistical analysis

The presented data are mean + standard deviation from 3 replicates. Statistical assessment employed Student’s t-test and one-way analysis of variance (ANOVA). Association between HC0074027, IGF1, and miR-379-5p mRNA was assessed with the Spearman rank correlation. All statistical analyses were performed using GraphPad Prism Software 7.0. P < 0.05 was the significance threshold.

## Results

In this study, we studied the biological role and the molecular mechanisms of HC0074027 in NSCLC. Our result indicated HC0074027 levels strongly correlate with NSCLC DTX-resistance. HC0074027 enhanced chemosensitivity of DRNCs *in vitro* and *in vivo*. Additionally, HC0074027 sponged miR-379-5p, and miR-379-5p targeted IGF1 to prevent chemoresistance in DRNCs. Thus, we revealed that HC0074027 modulates NSCLC chemoresistance via the miR-379-5p/IGF1 pathway, implying that our work highlighted a new underlying mechanism of NSCLC chemoresistance, which could be targeted to improve chemotherapy against NSCLC.

### HC0074027 levels strongly correlate with NSCLC DTX-resistance

**Figure 1.**
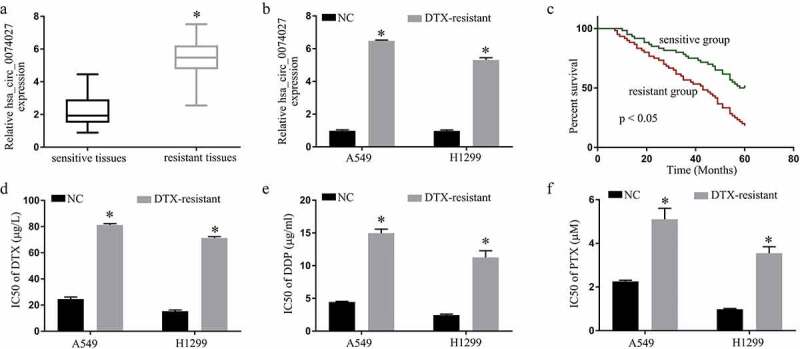
Hsa_circ_0074027 (HC0074027) levels are strongly correlated with NSCLC DTX-resistance. A. HC0074027 levels, via RT-qPCR, in sensitive and resistant tissues. B. HC0074027 levels, via RT-qPCR, in A549, H1299, A549/DTX, and H1299/DTX cells. C. The relationship between HC0074027 and overall survival of NSCLC patients. The IC50s of DTX (d), DDP (e), and PTX (f). The presented data are mean of 3 replicates, and *p < 0.05

HC0074027 levels were substantially elevated in DTX-resistant versus DTX-sensitive tissues and cells (Figure 1(a,b)). Patient clinicopathological characteristics are summarized in Table 1. The 5-year survival rate of the DTX-resistant patients, with elevated HC0074027 levels, was considerably lower, compared to the DTX-sensitive patients, with reduced HC0074027 levels (Figure 1(c)). We also demonstrated that the IC50s of DTX (Figure 1(d)), DDP (Figure 1(e)) and PTX (figure 1(f)) were high in the DDP-resistant cells verses normal cells.

**Table 1.** NSCLC clinicopathological features and hsa_circ_0074027 levels

Clinical and pathological characteristics	Cases	Hsa_circ_0074027 expression		P-value
		Low	High	
Age				0.854
< 60	61	32	29	
≥60	59	28	31	
Sex				0.716
Female	51	24	27	
Male	69	36	33	
TNM stage				< 0.05
I–II	62	42	20	
II–IV	58	18	40	
DTX				< 0.05
Sensitive	60	56	4	
Resistant	60	4	56	
Smoking status				0.679
Smoker	59	25	34	
Nonsmoker	61	35	26	
Lymph node metastasis				< 0.05
Yes	62	16	46	
No	58	44	14

### HC0074027 enhanced chemosensitivity of DRNCs

**Figure 2.**
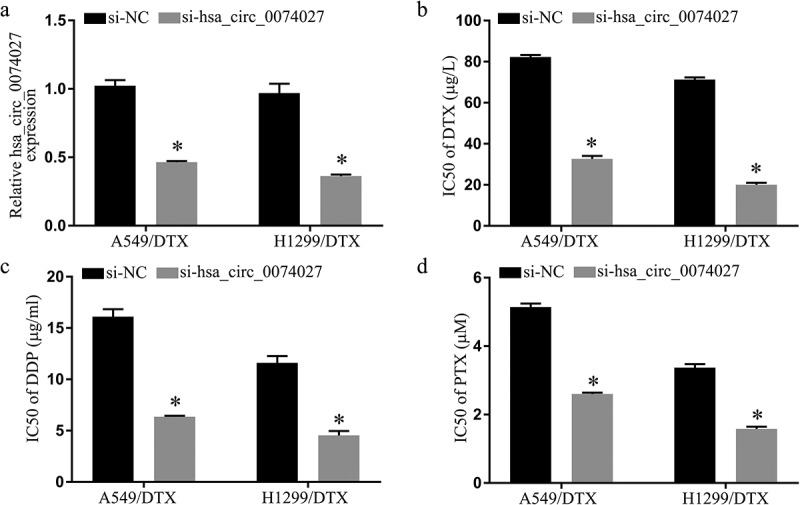
Hsa_circ_0074027 (HC0074027) downregulation inhibited chemoresistance in DTX-resistant NSCLC cells. A. HC0074027 quantification, via RT-qPCR, after silencing. IC50s of DTX (b), DDP (c), and PTX (d). The presented data are mean of 3 replicates, and *p < 0.05

To elucidate HC0074027 function in DRNCs, HC0074027 was silenced in both A-DTX and HN-DTX cells via siRNA incorporation. Based on our RT-qPCR data, si-HC0074027-incorporated cells exhibited markedly reduced HC0074027 expression, relative to control cells (Figure 2(a)). Moreover, the IC50s of DTX (Figure 2(b)), DDP (Figure 2(c)), and PTX (Figure 2(d)) displayed a significant reduction, compared to controls. Hence, there is a strong possibility that HC0074027 levels regulate NSCLC chemoresistance

### HC0074027 sponges miR-379-5p

**Figure 3.**
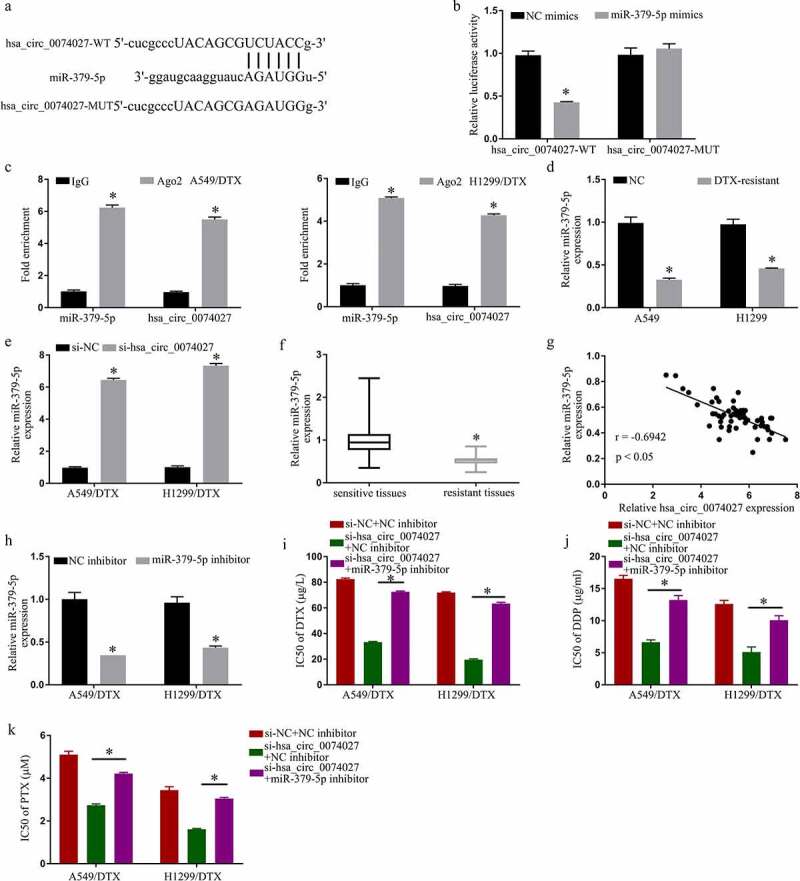
Hsa_circ_0074027 (HC0074027) sequesters miR-379-5p activity. A. Predicted docking sites of HC0074027 and miR-379-5p. B. Direct binding between HC0074027 and miR-379-5p, as evidenced by the DLR assay. C. RIP assay validated the HC0074027 interaction with miR-379-5p. D. MiR-379-5p levels, via RT-qPCR, in A549, H1299, A549/DTX, and H1299/DTX cells. E. MiR-379-5p levels, via RT-qPCR?, in DTX-resistant cells incorporated with si-NC, or si-hsa_circ_0074027. F. MiR-379-5p levels, via RT-qPCR, in sensitive and resistant tissues. G. Association between HC0074027 and miR-379-5p, as evidenced by the Spearman’s correlation coefficient. H. MiR-379-5p levels, via RT-qPCR, in DTX-resistant cells incorporated with NC-, or miR-379-5p-inhibitor. IC50s of DTX (i), DDP (j), and PTX (k). The presented data are mean of 3 replicates, and *p < 0.05

To examine the mechanistic pathway of HC0074027, we searched for potential miR docking sites within HC0074027. MiR-379-5p was identified to have sequence homology with HC0074027 (Figure 3(a)). To further confirm the HC0074027-miR-379-5p association, we carried out a DLR assay. Our analysis revealed that miR-379-5p-Ms drastically downregulated HC0074027-WT luciferase activity (Figure 3(b)). Furthermore, RIP analysis showed that HC0074027 and miR-379-5p were overtly enriched in the Ago2 group, as opposed to the lgG group (Figure 3(c)). Moreover, miR-379-5p levels in A-DTX and NH-DTX cells were significantly low, compared to A549 and H1299 cells (Figure 3(d)). Furthermore, HC0074027 depletion augmented miR-379-5p levels (Figure 3(e)). Additionally, compared to their sensitive counterparts, miR-379-5p levels were drastically reduced in DTX-resistant tissues (figure 3(f)). Moreover, the miR-379-5p levels were inversely proportional to HC0074027 levels (Figure 3(g)). Upon miR-379-5p-I incorporation, we demonstrated strong suppression of miR-379-5p levels (Figure 3(h)). Lastly, upon HC0074027 silencing, we demonstrated suppression of DTX (Figure 3(i)), DDP (Figure 3(j)) and PTX (Figure 3(k)) IC50s, whereas the low IC50 values were restored via miR-379-5p-I incorporation into A-DTX and NH-DTX cells.

### MiR-379-5p targets IGF1 to prevent chemoresistance in DRNCs

**Figure 4.**
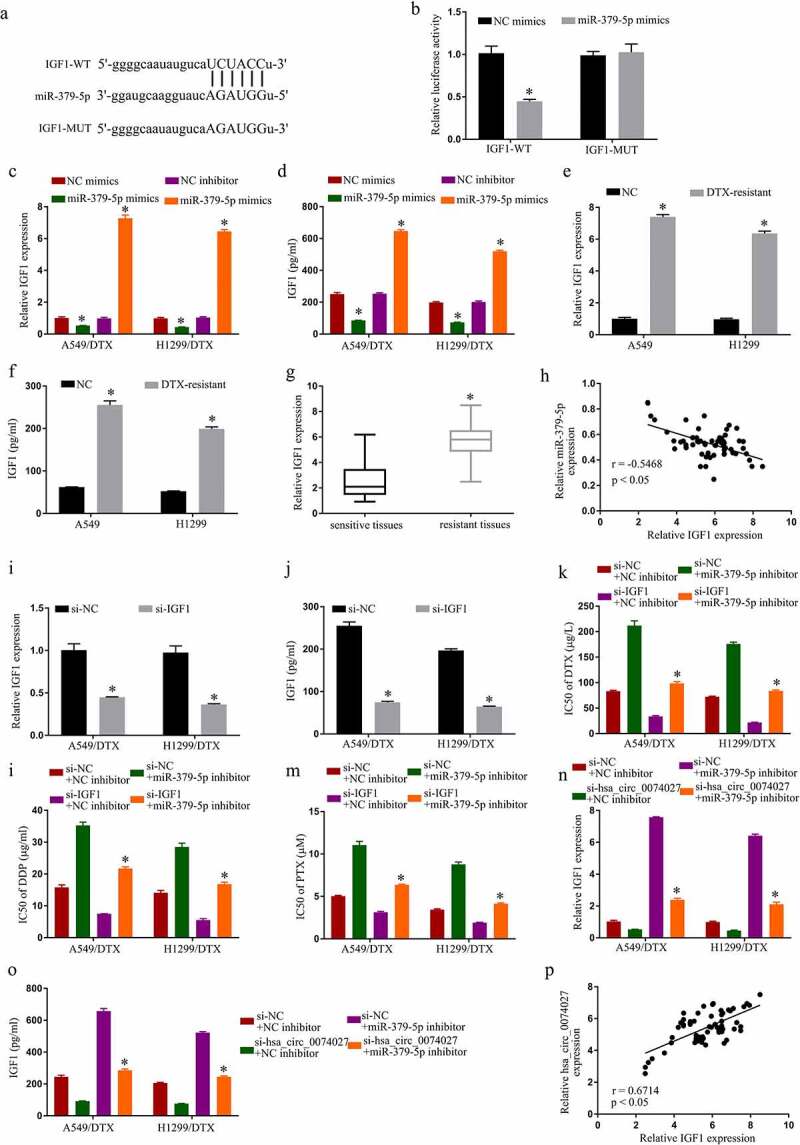
MiR-379-5p binds IGF1 and inhibits chemoresistance in DTX-resistant NSCLC cells. A. Predicted docking sites of IGF1 and miR-379-5p. B. IGF1 and miR-379-5p interaction validation, via DLR assay. IGF1 levels, via RT-qPCR (c) and Elisa assay (d). IGF1 levels, via RT-qPCR (e) and Elisa assay (f) in A549, H1299, A549/DTX, and H1299/DTX cells. G. IGF1 levels, via RT-qPCR, in sensitive and resistant tissues. H. IGF1 and miR-379-5p relationship, via the Spearman’s correlation coefficient. IGF1 levels, via RT-qPCR (i) and Elisa assay (j). IC50s of DTX (k), DDP (l), and PTX (m). IGF1 levels, via RT-qPCR (n) and Elisa assay (o). P. HC0074027 and IGF1 relationship, via the Spearman’s correlation coefficient. The presented data are mean of 3 replicates, and *p < 0.05

To identify miR-379-5p downstream target genes, we employed an online prediction software, which identified IGF1 as a target of miR-379-5p (Figure 4(a)). Using double luciferase detection, we further revealed that miR-379-5p-Ms decrease IGF1-WT activity (Figure 4(b)). Moreover, miR-379-5p-Ms incorporation into cells dramatically decreased levels of IGF1, and miR-379-5p-I incorporation increased it (Figure 4(c,d)). In addition, IGF1 levels in A-DTX and NH-DTX cells were considerably elevated, compared to A549 and H1299 cells (Figure 4(e,f)). Similarly, relative to the sensitive counterparts, IGF1 levels were markedly elevated in DTX-resistant tissues (Figure 4(g)). Moreover, IGF1 levels were inversely proportional to HC0074027 levels (Figure 4(h)). Additionally, upon si-IGF1 incorporation, IGF1 levels were severely suppressed (Figure 4(i,j)). MiR-379-5p silencing suppressed DTX (Figure 4(k)), DDP (Figure 4(l)), and PTX (Figure 4(m)) IC50s, and the low IC50 values were restored via si-IGF1 incorporation into A-DTX and NH-DTX cells. Moreover, miR-379-5p silencing reversed the suppression of HC0074027 silencing on IGF1 levels (Figure 4(n,o)). Lastly, HC0074027 levels were strongly correlated with IGF1 transcript levels (Figure 4(p)).

### *HC0074027 knockdown enhanced DTX sensitivity* in vivo

**Figure 5.**
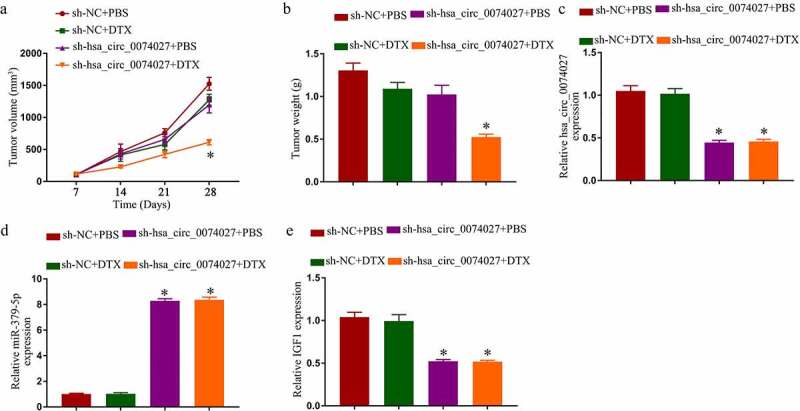
Hsa_circ_0074027 (HC0074027) downregulation induced DTX sensitivity *in vivo*. Tumor volume (a) and weight (b). HC0074027 (c), miR-379-5p (d), and IGF1 (e) levels, via RT-qPCR, in murine tumor tissues. The presented data are mean of 3 replicates, and *p < 0.05

We, next, tested HC0074027 regulation of DTX sensitivity in nude mice. Sh-HC0074027 transduction resulted in a strong suppression of tumor expansion, in presence or absence of DTX (Figure 5(a,b)). Surprisingly, DTX alone did not affect tumor expansion. However, a combined administration of DTX and sh-HC0074027 generated a strong suppression of tumor expansion (Figure 5(a,b)). Additionally, HC0074027 and IGF1 levels were markedly reduced and miR-379-5p levels were remarkably elevated in the sh-HC0074027-incorporated A-DTX tumors (Figure 5(c,d)).

## Discussion

Chemoresistance is the principal obstacle in cancer treatment [27]. Delineating the mechanism(s) behind drug resistance can help establish new targeted therapeutics that can efficiently suppress NSCLC progression. CircRNAs are well established chief modulators of NSCLC chemoresistance. Therefore, targeting relavant circRNAs may be a crucial step toward NSCLC resistance management. It is known that circ_0076305 modulates DDP NSCLC resistance via positive modulation of signal transducer and activator of transcription 3 (STAT3) via miR-296-5p sequestering [28]. Similarly, circ_0000079 serves as a decoy for the RNA-binding fragile X-related 1 (FXR1), preventing the assembly of the FXR1/ complex-partner protein kinase C, iota (PRCKI) complex, and reducing cell invasion and drug resistance in NSCLC [29]. Unfortunately, even though HC0074027 has long been established as a NSCLC oncogene [21–24], its role in NSCLC chemoresistance is unknown. We, therefore, examined the underlying mechanism affecting the HC0074027-mediated NSCLC resistance. We demonstrated that high HC0074027 levels correspond to DTX-resistance in NSCLC tissues and cells. Conversely, HC0074027 downregulation prevented NSCLC chemoresistance *in vitro* and *in vivo*.

MiR-379-5p is reportedly involved in the development of numerous cancers. LINC00665, for example, is known to accelerate breast cancer progression via the miR-379-5p/LIN28B pathway [30]. Alternately, miR-379-5p inhibits endometrial cancer cell proliferation and invasion via downregulation of RTK-like orphan receptor 1 (ROR1) levels [31]. Circ_SIPA1L1 induces osteosarcoma progression via the miR-379-5p/mitogen-activated protein kinase kinase kinase 9 (MAP3K9) pathway [32]. MiR-379-5p is a likely target of HC0074027, as the HC0074027 levels were previously shown to be inversely proportional to the anti-tumor activity of miR-379-5p in NSCLC [33]. Moreover, elevated miR-379-5p levels can reduce NSCLC chemoresistance. It was reported that circ_0011292 augments PTX resistance in NSCLC via modulation of the miR-379-5p/tripartite motif-containing protein 65 (TRIM65) pathway [18]. Based on our analysis, the miR-379-5p levels were strongly downregulated in the DTX-resistant NSCLC tissues and cells. Moreover, we verified a physical interaction between HC0074027 and miR-379-5p, which negatively affects miR-379-5p function. Hence, we propose that HC0074027 enhances NSCLC chemoresistance by sequestering miR-379-5p.

IGF1 expression is predominant in cancerous tissues like glioma [34] and colorectal cancers [35]. It is also prevalent in NSCLC and in small-cell lung cancer [36,37], and is known to contribute to NSCLC chemoresistance [38]. Herein, IGF1 levels were markedly elevated in DRNC and tissues. We are the first to report the miR-379-5p-mediated regulation of IGF1 activity, particularly in terms of NSCLC chemoresistance. Moreover, we also established a novel mechanism involving the HC0074027-miR-379-5p-IGF1 pathway. Based on these evidences, we propose that HC0074027 is an excellent candidate for targeted anti-NSCLC therapy.

## Conclusion

In summary, we revealed that HC0074027 modulates NSCLC chemoresistance via the miR-379-5p/IGF1 pathway. Our work highlights a new underlying mechanism of NSCLC chemoresistance, which can be targeted to improve chemotherapy against NSCLC.

## Highlights

1. Hsa_circ_0074027 suppressed NSCLC chemoresistance.
2. Hsa_circ_0074027 acted as a sponge of miR-379-5p.
3. Hsa_circ_0074027 promoted IGF1 expression by suppressed miR-379-5p.
4. Hsa_circ_0074027 promoted NSCLC chemoresistance via the miR-379-5p/IGF1 axis.
